# Microwave‐Assisted Efficient Intercalation for Fast Fabrication of High‐Quality and Large‐Size Single‐Layer Ti_3_C_2_T*_x_* Nanosheets

**DOI:** 10.1002/advs.202405686

**Published:** 2024-07-02

**Authors:** Yitian Zhong, Qixi Zhang, Shuling Lan, Haosheng Feng, Yanxi Zhao, Qin Li, Xianghong Li, Tao Huang

**Affiliations:** ^1^ Key Laboratory of Catalysis and Energy Materials Chemistry of Ministry of Education College of Chemistry and Materials Science South‐Central Minzu University Wuhan 430074 China

**Keywords:** intercalation, microwave, MXene, nanosheets, Ti_3_C_2_T*_x_*

## Abstract

A novel microwave‐assisted intercalation (MAI) strategy is proposed for fast and efficient intercalation of layered MXene to prepare large‐size single‐layer MXene. After LiF‐HCl etching of Ti_3_AlC_2_, the as‐prepared multi‐layer Ti_3_C_2_T*_x_* (M‐T) are intercalated with Li_3_AlF_6_ as an intercalator and ethylene glycol (EG) as a solvent under microwave irradiation for 5 min. Furthermore, the dispersed high‐quality large‐sized single‐layer Ti_3_C_2_T*_x_* (S‐T) nanosheets with a thickness of 1.66 nm and a large lateral size over 20 µm are achieved with a yield of over 60% after a further ultrasonic delamination followed by electrostatic precipitation, acid washing, and calcination. In addition, Pd/S‐T composite catalyst, which is constructed with Pd nanoparticles supported on the as‐prepared S‐T nanosheets, exhibits an excellent performance for rapid and efficient selective hydrogenation of nitroarenes with H_2_ under a mild condition. At room temperature, full conversion of nitrobenzene and 100% aniline selectivity are achieved over Pd/S‐T catalyst in 20 min with 0.5 MPa of H_2_ pressure. This work provides a novel method for facile, fast, and large‐scale preparation of single‐layer MXene and develops a new approach for constructing efficient nanocatalytic systems.

## Graphical abstract

A novel microwave‐assisted intercalation (MAI) strategy is proposed for efficient intercalation of layered MXene to prepare large‐sized single‐layer MXene. After intercalation of multi‐layer Ti_3_C_2_T*_x_* (M‐T) with Li_3_AlF_6_ as an intercalator and ethylene glycol as a solvent under microwave irradiation for 5 min, high‐quality large‐sized single‐layer Ti_3_C_2_T*_x_* nanosheets (S‐T) are achieved finally by a series simple treatments.

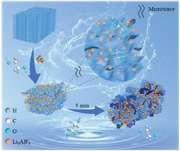

## 1 Introduction

As a novel family of 2D carbides and nitrides, MXenes with a general chemical formula of M*_n_*_+1_X*_n_* T*_x_* (M stands for a transition metal, X is C or N, T*_x_* stands for surface functional groups such as ─OH, ─NH_2_, ─F or ─O terminations, *n* = 1, 2, or 3) have been paid much attention in recent years because of their excellent electronical conductivity, hydrophilicity, mechanical strength, porsity and stability as well as their potential applications in the field of energy storage and catalysis.^[^ ^1^, ^2^, ^3^, ^4^, ^5^, ^6^, ^7^, ^8^, ^9^, ^10^ ^]^ Generally, MXene can be obtained by selective etching of MAX phase, a new type of ternary layered compound with a general formula M*_n_*_+1_AX*_n_* in which the interleaving layers A (IIIA or IVA elements) can be chemically extracted.^[^ ^1^, ^2^, ^3^, ^4^, ^5^, ^6^, ^7^, ^8^, ^9^, ^10^, ^11^, ^12^, ^13^, ^14^, ^15^ ^]^ Currently, a large‐scale efficient preparation of MXenes is still receiving more attention for their important applications.

In the MXene family, so far, Ti_3_C_2_T*_x_* is the most representative 2D transition metal carbides due to its advantages of low cost, simple preparation process, and rich and controllable surface functional groups.^[^ ^16^, ^17^ ^]^ At present, the preparation of Ti_3_C_2_T*_x_* flakes can be prepared by etching the Al interleaving layers of titanium aluminum carbide (Ti_3_AlC_2_), which is one of the most common MAX phase materials, with concentrated hydrofluoric acid (HF).^[^ ^1^, ^2^, ^18^, ^19^ ^]^ However, it often consumed a large amount of toxic and highly corrosive HF with a certain hazard.^[^ ^20^ ^]^ Alternatively, an etching solution composed of LiF and HCl to form HF in situ was used and the intercalation effect of residual Li^+^ ions during the etching process resulted in spontaneous stratification, generating Ti_3_C_2_T*_x_* with accordion‐like layered structures.^[^ ^21^ ^]^ The compact structure of multi‐layer MXene and the strong Van der Waals forces between layers lead to inevitable self‐stacking of MXene. Though the accordion‐like Ti_3_C_2_T*_x_* stack with an appropriate interlayer spacing can facilitate the effective electron transport, it results in a low availability of a large number of redox active sites, which restricted its applications in batteries, catalysis, and other fields. In addition, the yield of thin layer Ti_3_C_2_T*_x_* prepared direct HF etching was often lower than 20% because of the strong interaction between potential Ti─Ti bonds and the residual Ti ─ bonds between adjacent Ti_3_C_2_T*_x_* layers, which hindered effective intercalation and further mechanical delamination.^[^ ^20^ ^]^ Some organic molecules such as isopropylamine, dimethylformamide (DMF), dimethyl sulfoxide (DMSO), ammonium hydroxide (TBAOH), tetramethylammonium hydroxide (TMAOH), etc. were generally employed as intercalators to intercalate the multilayered sheets.^[^ ^22^, ^23^, ^24^, ^25^ ^]^ After intercalation with stirring for a certain time, delaminated thin‐layered Ti_3_C_2_T*_x_* were obtained by ultrasonication.^[^ ^26^, ^27^, ^28^ ^]^ Han et al. proposed an efficient method based on hydrothermal‐assisted intercalation to improve the yield of MXene, achieving a high yield of 74%.^[^ ^29^ ^]^ In addition, Huang et al. proposed a freezing‐and‐thawing (FAT) approach to exfoliate multilayer‐MXene using the volume expansion of intercalated water and the yield of large FAT‐MXene flakes with special wrinkles can reach 39% after four cycles of the FAT process.^[^ ^30^ ^]^ However, these methods have disadvantages such as complex steps, long preparation times, and high energy consumption as well as limited size, low delamination, shredded pieces or oxidation of MXene,^[^ ^31^, ^32^, ^33^ ^]^ seriously reducing its superior performance in applications. So, efficient and fast fabrication of high‐quality and large‐size single‐layer Ti_3_C_2_T*_x_* nanosheets is imperative and remains a challenge.

In this paper, a novel microwave‐assisted intercalating (MAI) strategy for fast intercalation of layered MXene was proposed to fabricate large‐sized single‐layer Ti_3_C_2_T*_x_*. Microwave irradiation has been widely used in various reactions due to its advantages of deep, fast and uniform heat as well as convenience, controllability, energy conservation and high efficiency. Microwave irradiation may be favorable for organic molecule‐assisted intercalation of layered MXene by accelerating the vibration, rotation and resonance of polar molecules.^[^ ^34^, ^35^, ^36^, ^37^ ^]^ Herein, after LiF‐HCl etching of Ti_3_AlC_2_, the as‐prepared layered Ti_3_C_2_T*_x_* particles were intercalated with Li_3_AlF_6_ as an intercalator and ethylene glycol (EG) as a solvent. Then, after a further delamination promoted by subsequent short‐term ultrasonication, precipitation with the action of NH_4_^+^ ions, acid washing, freeze‐drying and annealing successively, high‐quality and large‐size single‐layer Ti_3_C_2_T*_x_* nanosheets were obtained with a yield of over 60%. The reaction parameters of MAI were systematically investigated. Furthermore, Single layer Ti_3_C_2_T*_x_* can serve as a potential candidate for loading metal nanoparticles for catalytic applications due to a large specific surface area and abundant surface groups.^[^ ^38^ ^]^ Ti_3_C_2_T*_x_* as a substrate may be favorable for the in situ growth of metal nanoparticles and prevent the agglomeration of nanocatalysts.^[^ ^39^ ^]^ Therefore, a novel efficient nanocatalytic system was constructed with Pd nanoparticles supported on the as‐prepared single layer Ti_3_C_2_T*_x_* nanosheets and its performance for efficient and selective hydrogenation of nitroaromatic compounds was investigated.

## 2 Results and Discussion

**Figure 1:**
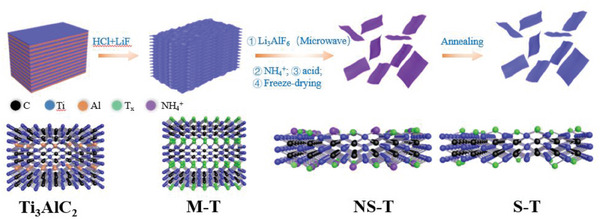
The schematic diagram of the S‐T preparation.

The experimental procedure and schematic diagram for the preparation of the single‐layer Ti_3_C_2_T*_x_* nanosheets (S‐T) are shown in Figure S1, Supporting Information and **Figure** **1**. Initially, after etching of Ti_3_AlC_2_ by HF generated in situ with HCl and LiF under hydrothermal conditions, the multi‐layer Ti_3_C_2_T*_x_* (M‐T) black powders were obtained (Figure S1, Supporting Information). Generally, the unexcavated Ti_3_AlC_2_ exhibits a compact layered structure (Figure 1). After etching off the Al layers, M‐T with an increased interlayer spacing shows an accordion‐like structure with some surface functional groups T*_X_*. By means of MAI strategy, M‐T can be well intercalated into single layer sheets under microwave irradiation. After ultrasonic treatment for the intercalated M‐T with Li_3_AlF_6_ (donated as L‐T), the collected upper suspension showed clear Tyndall effect (Figure S2a, Supporting Information), indicating good dispersibility and hydrophilicity for the L‐T sample. Under the action of NH_4_^+^ ions, the negatively charged single‐layer Ti_3_C_2_T*_x_* nanosheets were precipitated as black floccules due to electrostatic flocculation (Figure S2b,c, Supporting Information). Followed by acid washing and freeze‐drying successively, the fluffy black powdery single‐layer Ti_3_C_2_T*_x_* nanosheets (NS‐T) with ammonium ions adsorbed on the surfaces were obtained. It was noteworthy that, after annealing to removing the adsorbed NH_4_^+^ ions, the as‐obtained black single‐layer Ti_3_C_2_T*_x_* nanosheets (S‐T) appeared with lithe, fluffy and cottony nature (Figure S2d, Supporting Information). As a comparison, the volume of 25 mg S‐T showed much larger than that of 50 mg M‐T (the inset of Figure S2d, Supporting Information), suggesting a high exfoliation of the as‐prepared single layer Ti_3_C_2_T*_x_* nanosheets.

**Figure 2:**
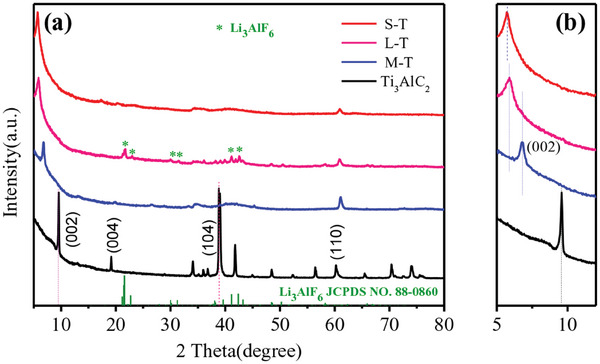
XRD patterns of Ti_3_AlC_2_, M‐T, L‐T and S‐T samples. a) Full range. b) Corresponding partial zoom.

**Figure** **2**a shows the XRD spectra of Ti_3_AlC_2_, M‐T, L‐T and S‐T samples. Generally, the diffraction peaks at 9.4° and 39° should be indexed to (002) and (104) planes of Ti_3_AlC_2_, respectively. The disappearance of the peak at 39° in M‐T sample indicated the Al layers have been etched completely, while the shift of the peak at 9.4° to 6.8° should be attributed to the removal of Al layers and the introduction of termination groups. Furthermore, after microwave‐assisted intercalation for the M‐T sample, the corresponding (002) crystal plane of Ti_3_C_2_T*_x_* in L‐T samples shifted towards a small angle (Figure 2b), which may be ascribed to a significant increase of interlayer spacing due to the insertion of Li_3_AlF_6_ into the interlayers of Ti_3_C_2_T*_x_* under microwave irradiation. Meanwhile, some characteristic peaks of the intercalating agent (Li_3_AlF_6_, JCPDS NO.88‐0860) appeared in L‐T samples, indicating the existence of Li_3_AlF_6_. After ultrasonic‐assisted exfoliation, the (002) crystal plane of Ti_3_C_2_T*_x_* in S‐T further shifted towards small angle, implying a further delamination of single layer Ti_3_C_2_T*_x_*.

With 50 mg of M‐T, the yield of the as‐obtained S‐T was 63.4% when the molar of intercalating agent Li_3_AlF_6_ was 0.3 mmol (Figure S3, Supporting Information), whereas a lower yield (47.8%) of S‐T was obtained with using 0.1 mmol of Li_3_AlF_6_ due to incomplete intercalation. An excessive intercalating agent also reduced intercalation efficiency (50.7%) due to blocked insertion of Li_3_AlF_6_.

**Figure 3:**
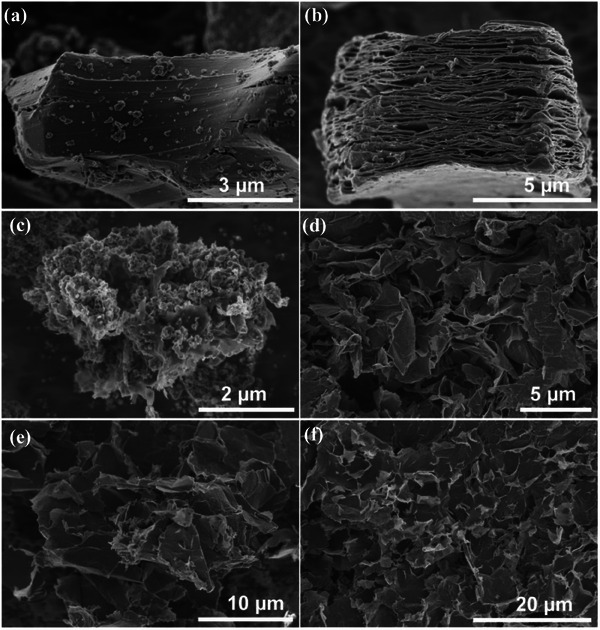
SEM images of different samples. a) Ti_3_AlC_2_. b) M‐T. c) L‐T. d) L‐T‐acid. e) NS‐T. f) S‐T.

Compared with the unetched layered Ti_3_AlC_2_ (**Figure** **3**a), after selectively etching of the Al layers, accordion shaped multi‐layer Ti_3_C_2_T*_x_* samples with some small rod‐shaped particles adsorbed on their surfaces were obtained (Figure S4, Supporting Information). The corresponding XRD pattern showed the characteristic peaks according to Li_3_AlF_6_, confirming that these particles should be assigned to the by‐product Li_3_AlF_6_ generated in the etching process (Figure S5, Supporting Information). After acid washing with dilute sulfuric acid, accordion shaped multi‐layer Ti_3_C_2_T*_x_* (M‐T) samples with smooth surfaces were obtained (Figure 3b). Moreover, after microwave irradiation for 5 min in EG without adding any intercalator, it was found that the interlayer spacing increased significantly for the uncleaned accordion‐shaped multi‐layer Ti_3_C_2_T*_x_* obtained by etching (Figure S6a, Supporting Information), while no obvious change in the interlayer spacing were observed for the M‐T samples with clean surfaces under the same conditions (Figure S6b, Supporting Information). These results indicated that the by‐product Li_3_AlF_6_ presented an intercalation effect under microwave irradiation. Therefore, the microwave‐assisted intercalation (MAI) strategy by adding Li_3_AlF_6_ as an intercalating agent was proposed.

By MAI strategy with introducing extrinsic Li_3_AlF_6_ as an intercalator for an intercalation of M‐T sample, the interlayer spacing was further swelled up owing to the insertion of Li_3_AlF_6_ particles (Figure 3c), which corresponded to L‐T sample, although the sheet structure looked unclear due to the coverage of Li_3_AlF_6_ particles. As shown in Figure 3c, a large amount of Li_3_AlF_6_ particles were embedded between Ti_3_C_2_T*_x_* layers and adsorbed on their surfaces. After treatment of L‐T sample by acid washing to removing Li_3_AlF_6_, the accordion‐like structures disappeared and turned into stacked single‐layer nanosheets with clean and smooth surfaces (donated as L‐T‐acid) which were twisted due to their ultrathinness (Figure 3d). Thus, the intercalation of Li_3_AlF_6_ overcome the Van der Waals forces between interlayers of Ti_3_C_2_T*_x_*. In order to better promote flocculation of single layer Ti_3_C_2_T*_x_* nanosheets, ammonium salt was added directly into L‐T sample and then acid washing to remove Li_3_AlF_6_ followed by freeze‐drying, and NS‐T sample was generated (Figure 3e). Furthermore, ultrathin single layer Ti_3_C_2_T*_x_* nanosheets (S‐T sample) without surface ammonium ions were obtained after annealing (Figure 3f), presenting a high quality and yield although still in significant wrinkles. Especially, the resulting S‐T sample presented a large size over 20 µm within planar extension (Figure S7, Supporting Information). In addition, TEM observation also displayed the large‐sized and ultrathin single‐layer feature of S‐T sample (Figure S8, Supporting Information). This may be attributed to the microwave‐assisted rapid intercalation as well as a short time ultrasonic exfoliation.

In the etching reaction, LiCl was also produced besides Li_3_AlF_6_. So, to further investigate the role of Li_3_AlF_6_ upon the intercalation of the clean multi‐layer Ti_3_C_2_T*_x_* (M‐T sample) by MAI strategy, LiCl, KCl or KCl+Li_3_AlF_6_ was used as an intercalator instead of single Li_3_AlF_6_ as a contrast, respectively. The XRD patterns and the corresponding SEM images of the final products obtained using different intercalators followed by ultrasonic exfoliation were shown in Figures S9 and S10, Supporting Information. Compared with that of the M‐T sample with introducing no intercalator, the (002) crystal planes of the obtained samples shifted significantly to low angles by either Li_3_AlF_6_ or KCl+Li_3_AlF_6_ intercalating approach, indicating an increase in the interlayer spacing as well as a good intercalation effect. With only LiCl or KCl as an intercalator, however, no obvious change was observed for the (002) crystal plane position, indicating poor intercalation effect with using LiCl or KCl. Therefore, Li_3_AlF_6_ played a good intercalating role by MAI strategy, while Li^+^ or Cl‾ ions had no intercalation effect. Correspondingly, as can be seen in Figure S10a,b, Supporting Information, the layered structure still maintained with using either LiCl or KCl as an intercalating agent, indicating no intercalation effect, which was similar to that of M‐T sample without any intercalator after microwave irradiation (Figure S6b, Supporting Information). Nevertheless, single layer Ti_3_C_2_T*_x_* ultrathin nanosheets same as S‐T were produced for KCl+Li_3_AlF_6_ intercalating system (Figure S10c, Supporting Information) compared with Li_3_AlF_6_ intercalating system (Figure S10d, Supporting Information). These results showed that Li_3_AlF_6_ was the best one of intercalators to achieve good intercalation effect, while the presence of LiCl or KCl played no role in the intercalation of multi‐layer Ti_3_C_2_T*_x_*, which were consistent with the corresponding XRD observations.

The accordion‐like structures of the M‐T sample with compacted interlayer spacing can also be observed in TEM (Figure S11a, Supporting Information). After intercalation by Li_3_AlF_6_, M‐T was turned to L‐T and TEM observation presented comparatively dispersed single layer nanosheets (Figure S11b, Supporting Information). The EDS elemental mappings corresponding to HAADF‐STEM image (Figure S11c_1_, Supporting Information) revealed C and Ti elements with uniform distribution for the L‐T samples, as shown in Figure S11c_2_,c_3_, Supporting Information, respectively. Meanwhile, F, Al and Cl elements were also identified, as shown in Figures S11c_4_,c_5__,_ and S6c_6_, Supporting Information, respectively. The elemental composition analyses displayed F of 6 times that of Al (Figure S11d, Supporting Information), confirming a successful intercalation of Li_3_AlF_6_. In addition, a very small amount of Cl element resulted from the etchant HCl was detected.

**Figure 4:**
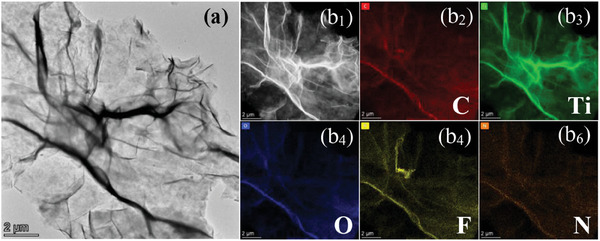
Characterization of the NS‐T monolayer after acid washing followed by freeze‐drying. a) TEM image. b_1_) HAADF‐STEM image; b_2_–b_6_) EDS mappings of the main composition elements C (b_2_, red), Ti (b_3_, green), O (b_4_, blue), F (b_5_, yellow) and N (b_6_, brown), respectively.

Especially, the wrinkled surface of NS‐T monolayer obtained with NH_4_^+^ electrostatic flocculation followed by acid washing and freeze‐drying was also observed in TEM (**Figure** **4**a). The EDS elemental mappings of NS‐T monolayer corresponding to HAADF‐STEM image (Figure 4b_1_) revealed uniform distribution of C (b_2_, red), Ti (b_3_, green), O (b_4_, blue) and F (b_5_, yellow) elements, as well as a few element N (b_6_, brown) originated from NH_4_^+^ ions. The corresponding elemental composition analyses were shown in Figure S11e, Supporting Information. Compared with L‐T sample (Figure S11d, Supporting Information), the disappearance of Al in NS‐T sample (Figure S11e, Supporting Information) confirmed that Li_3_AlF_6_ has been removed except for the residual F groups on the surfaces after acid washing with dilute sulfuric acid. Furthermore, the EDS elemental mappings of S‐T sample revealed the existence of C (red), Ti (green), O (blue) and F (yellow) elements with the exception of N element (Figure S12, Supporting Information), confirming the disappearance of N in S‐T sample obtained by annealing of NS‐T.

Zeta potential of Li_3_AlF_6_ was measured to be −8.77 mV, showing a weak repulsive force between Li_3_AlF_6_ particles, while a higher Zeta potential of −21.70 mV was observed for M‐T sample, confirming abundant negatively‐charged functional groups on Ti_3_C_2_T*_x_* surfaces as well as relatively strong interlayer repulsion (Figure S13, Supporting Information). Moreover, Zeta potential of the as‐prepared L‐T sample reached −26.79 mV, which revealed an increased surface electronegativity and enhanced interlayer repulsion due to Li_3_AlF_6_ intercalation for Ti_3_C_2_T*_x_*. As a result, the interlayer spacing of L‐T significantly increased. Thus Li_3_AlF_6_ could indeed take a good intercalation effect.

**Figure 5:**
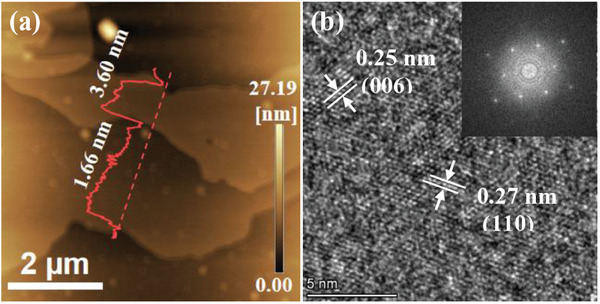
a) AFM image and height cutaway view of S‐T. b) HRTEM image of S‐T. The inset of (b) is FFT pattern.

Atomic force microscopy (AFM) images and height cutaway views showed that the monolayer thickness of Ti_3_C_2_T*_x_* nanosheets was about 1.66 nm and the double‐layer thickness was about 3.60 nm (**Figure** **5**a). Both of the 2D and 3D AFM images showed uniform topography as well as ultrathin thickness of monolayer Ti_3_C_2_T*_x_* nanosheet (Figure S14, Supporting Information). The HRTEM image and the corresponding fast Fourier transform (FFT) pattern (the inset) (Figure 5b) as well as the selected area electron diffraction (SAED) (Figure S15, Supporting Information) demonstrated a single crystal structure and good crystallinity of the large‐sized monolayer Ti_3_C_2_T*_x_*. The lattice spacing of 0.25 and 0.27 nm can be indexed to the (006) and (110) crystal plane of the large‐sized monolayer Ti_3_C_2_T*_x_*, respectively, indicating its hexagonal crystal structure.

**Figure 6:**
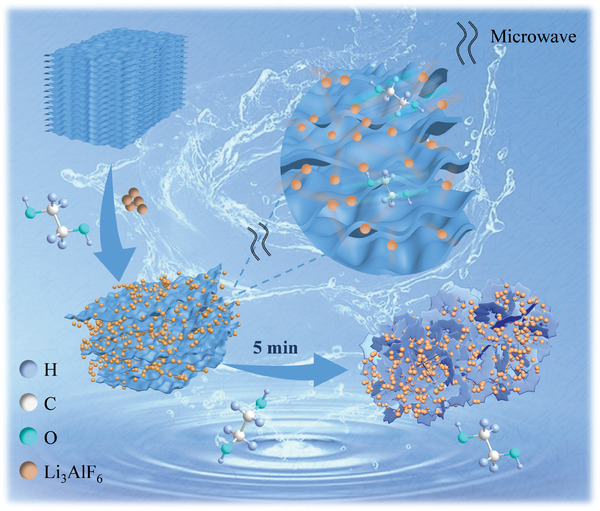
Schematic illustration of the intercalation mechanism by MAI strategy.

According to the above results, a schematic illustration of the intercalation mechanism by MAI strategy can be described as **Figure** **6**. EG was used as a polar solvent and an antioxidant to adsorb microwave energy. Under microwave irradiation, the granular intercalating agent Li_3_AlF_6_ moved quickly along with the solvent molecules and penetrated into interlayers, which led to an increase in surface electronegativity and interlayer repulsion. As a result, multi‐layer Ti_3_C_2_T*_x_* (M‐T) were intercalated through a violent vibration and collision between Li_3_AlF_6_ and Ti_3_C_2_T*_x_* layers, and ultrthin single‐layer sheets were generated.

Furthermore, the surface element composition and chemical bonds in S‐T sample were further investigated by XPS measurements. The full‐scale XPS spectrum presented Ti, C, O and F elements in S‐T sample was shown in Figure S16, Supporting Information. The characteristic peaks of Ti 2p region in the fine spectra showed two parts of Ti 2p_3/2_ and Ti 2p_1/2_ (Figure S17a, Supporting Information). XPS‐peak‐differentiating analyses for Ti 2p_3/2_ demonstrated three peaks at 455.4, 456.5, and 459.5 eV, corresponding to Ti─C, Ti─F*_X_* and Ti─O bonds, respectively, whereas three peaks at 461.6, 462.5 and 465.0 eV for Ti 2p_1/2_ were attributed to Ti─C, Ti─O and Ti─F*_X_* bonds, respectively. XPS‐peak‐differentiating analyses for C 1s region showed the binding energies at 281.9, 284.9, 286.3 and 288.9 eV (Figure S17b, Supporting Information), corresponding to Ti─C, C─C, C─O and C═O bonds, respectively. The binding energies of O 1s at 530.0, 532.2, 685.2 eV (Figure S17c, Supporting Information) were attributed to Ti─O, O─C─(OH)*_X_* and C─Ti─F*_X_* bonds, respectively, and the binding energy of F 1s at 685.2 eV was attributed to C─Ti─F*_X_* bonds (Figure S17d, Supporting Information), indicating the existence of ─O, ─OH, and ─F terminations on the surfaces of S‐T samples.

**Figure 7:**
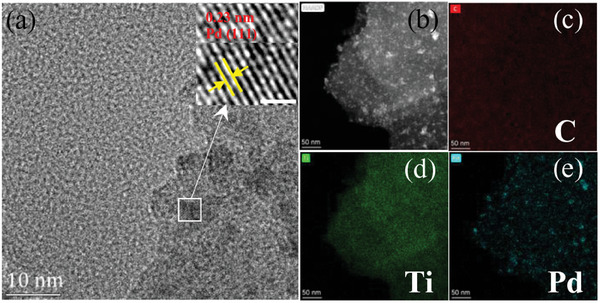
a) HRTEM images of Pd/S‐T. b) HAADF‐STEM image of Pd/S‐T. c–e) EDS mappings of C, Ti and Pd elements, respectively.

According to the literature,^[^ ^40^ ^]^ abundant terminal groups on the surface of MXene would facilitate the adsorption of metal precursors and Ti vacancy defects may be favorable for anchoring metal nanoparticles. Ti vacancy defects on the surface of the as‐prepared S‐T sample could be observed by HREM observation, as shown in Figure S18, Supporting Information. Therefore, S‐T sample was employed as substrate to construct MXene‐loaded Pd nanoparticles. After alkaline treatment, a large amount of Pd nanoparticles were anchored in situ on the surface of S‐T. The SEM image of the as‐prepared Pd/S‐T composite was shown in Figure S19, Supporting Information. The TEM and HRTEM observations confirmed a successful loading of Pd nanoparticles with an average size of 3.65 nm and a lattice spacing of 0.23 nm which was indexed to the Pd (111) crystal planes (**Figure** **7**a and Figure S20, Supporting Information). As can be seen, Ti_3_C_2_T*_x_* still maintained a layered structure though covering Pd nanoparticles with small sizes and uniform distribution. Moreover, with the successful loading of Pd nanoparticles, the surface of Ti_3_C_2_T*_x_* became flat without wrinkles though somewhat rough. Meanwhile, the EDS elemental mappings corresponding to HAADF‐STEM image (Figure 7b) revealed uniform distribution of C, Ti and O elements (Figure 7c–e). Thus, the large surface of S‐T was favorable for the loading of Pd nanoparticles. The XRD pattern of Pd/S‐T composite showed two weak characteristic peaks of Pd (111) and (200) crystal planes at 39.1 and 45.4° of 2*θ* (Figure S21a, Supporting Information). The full‐scale XPS spectra of Pd/S‐T sample also presented Pd besides Ti, C, O and F elements compared with that of S‐T sample (Figure S21b, Supporting Information). The fine XPS spectra of Pd/S‐T showed the binding energies of Pd 3d_5/2_ and Pd 3d_3/2_ at 335.0 and 340.3 eV (Figure S21c, Supporting Information), respectively, in addition to those of Ti 2p, C 1s and O 1s regions (Figure S21d–f, Supporting Information). The binding energies at 341.0 and 335.9 eV by XPS‐peak‐differentiating analysis should be assigned to PdO*_X_* (Figure S21c, Supporting Information), which may be ascribed to minor oxidation of Pd.

The catalytic performance of Pd/S‐T composite was investigated by the hydrogenation of nitrobenzene and its derivatives. The Pd loading in Pd/S‐T composite was determined to be 6.89 wt% by ICP‐OES. The hydrogenation reaction was carried out with 1.0 mmol of nitrobenzene in the presence of 5 mg of Pd/S‐T as catalyst (0.32 mol% Pd relative to nitrobenzene) and 10 ml of ethanol as a solvent under a H_2_ pressure of 0.5 MPa at room temperature (25 °C). As a contrast, the same experiment was conducted using Pd/C catalyst with an equal amount of Pd. With increasing the reaction time, nitrobenzene was converted into aniline via the phenylhydroxylamine intermediate. It was worth noting that both the conversion of nitrobenzene and the selectivity of aniline reached 100% after reaction for 20 min over Pd/S‐T, as shown in Figure S22 and Table S1 (entry 1), Supporting Information, while 87.5% of conversion and 97.7% of aniline selectivity were achieved over Pd/C catalyst Figure S23, Supporting Information. Besides nitrobenzene, Pd/S‐T composite catalyst also afforded complete conversion and >99% aniline selectivity at room temperature for other substituted nitrobenzene with electron donating groups (i.e., ─CH_3_ or ─C_2_H_5_, Table S1, entries 2 and 3, Supporting Information), while for nitrobenzene derivatives with electron withdrawing groups (i.e., ─F or ─CHO, Table S1, entries 4 and 5, Supporting Information), an increase in the amount of catalyst and the reaction time would be made to achieve a complete conversion and >99% aniline selectivity. The GC analyses of hydrogenation products of the corresponding nitrobenzene derivatives were shown in Figure S24, Supporting Information.

**Figure 8:**
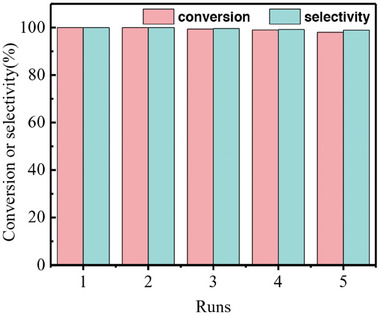
Recyclability of Pd/S‐T catalyst for five cycles of nitrobenzene hydrogenation.

The reusability and stability of Pd/S‐T composite catalyst was examined by nitrobenzene hydrogenation. After the reaction, a small amount of supernatant was extracted and filtered with microporous filter membrane for GC detection. Then 1 mmol of fresh substrate was added for the next run under identical conditions. As shown in **Figure** **8** and Figure S25, Supporting Information, 98% nitrobenzene conversion and 99% aniline selectivity were maintained after five cycles. A slight decrease in conversion rate and selectivity should be ascribed to a little loss of the catalyst during the extracting process of products. In addition, TEM and SEM measurements for the Pd/S‐T composite catalyst after 5 cycles showed that the layered structure covering with uniform and dispersed Pd nanoparticles remained almost the same except for a slight increase in the size of Pd nanoparticles (Figure S26, Supporting Information). These results demonstrated a high reusability and stability of Pd/S‐T composite catalyst.

**Figure 9:**
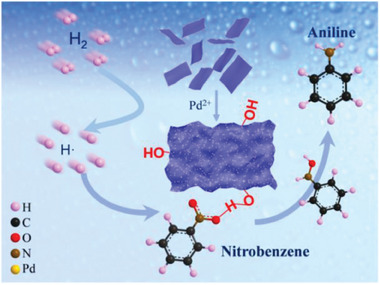
Schematic diagram of nitrobenzene hydrogenation over Pd/S‐T catalyst.

According to the GC analyses above, the hydrogenation of nitrobenzene over Pd/S‐T catalyst followed a pathway via phenylhydroxylamine intermediate. During the hydrogenation process, on the one hand, the zero valent Pd on the surfaces of Pd/S‐T sheets facilitated the adsorption of H_2_ followed by dissociating to active hydrogen, which would violently attack the nitro group of the substrate. On the other hand, hydrogen bonds can be formed between nitrobenzene and Pd/S‐T catalyst by means of nitro and the surface ─OH groups, which was favorable for the adsorption of nitrobenzene on the surface of catalyst and promoted the activation of the nitro groups and the formation of phenylhydroxylamine intermediates. As a result, hydrogenation activity and selectivity were improved in a short time at room temperature. The mechanism for nitrobenzene hydrogenation can be illustrated as Figure S27, Supporting Information, as well as the schematic diagram (**Figure** **9**).

## 3 Conclusion

In summary, a novel MAI strategy with Li_3_AlF_6_ as an intercalator and EG as a solvent was proposed for fast intercalation of layered MXene to prepare large‐size single‐layer Ti_3_C_2_T*_x_* nanosheets. Under microwave irradiation for 5 min, the intercalating agent Li_3_AlF_6_ accompanying with solvent molecules was pushed into the interlayers of the multi‐layer Ti_3_C_2_T*_x_* (M‐T) and then the multi‐layer structures were delaminated due to the intercalation of Li_3_AlF_6_. After further ultrasonic treatment, stacked single‐layer nanosheets with wrinkles were obtained. After electrostatic precipitation, acid washing, freeze‐drying and annealing, the dispersed single layer Ti_3_C_2_T*_x_* nanosheets (S‐T) with a thickness of 1.66 nm and a large planar size over 20 µm were achieved. Compared with traditional hydrothermal methods driving slow intercalation of organic molecules, the MAI strategy is more effective for the fabrication of high‐quality large‐size single layer Ti_3_C_2_T*_x_* nanosheets. The S‐T nanosheets were used as a carrier to load uniform and monodispersive metal Pd nanoparticles. The as‐prepared Pd/S‐T composite exhibited an excellent performance for rapid and efficient selective hydrogenation of nitroarenes with H_2_ under a mild condition. At room temperature, full conversion of nitrobenzene and 100% aniline selectivity were achieved over Pd/S‐T composite catalyst in 20 min with 0.5 MPa of H_2_ pressure. Meanwhile, Pd/S‐T composite catalyst demonstrated a high reusability and stability. Therefore, this work developed a microwave‐assisted fast intercalation strategy for rapid and large‐scale preparation of single‐layer MXene and also demonstrated a promising application prospect of Ti_3_C_2_T*_x_* MXene in the field of catalysis

## 4 Experimental Section

### Materials

Ti_3_AlC_2_ (400 mesh, ≥98%) was purchased from Jilin 11 Technology Co., Ltd., China. Li_3_AlF_6_ (≥98%) was purchased from Shanghai Maklin Biochem. Co., Ltd.,China. PdCl_2_ was purchased from SINO‐Platinum Materials Co., Ltd., China. EG, LiF (≥99.9%), HCl (36‐38%), LiCl, KCl, NH_4_HCO_3_, KOH, NaBH_4_, H_2_SO_4_, ethylene glycol (EG), anhydrous ethanol and hexadecyltrimethylammonium bromide (CTAB) were purchased from Sinopharm Chemical Reagent Co., Ltd. All reagents were analytical purity and used without further purification. Deionized water was used in all experiments.

### Syntheses of Multi‐Layer Ti_3_C_2_T*_x_*

In a typical synthesis process, 1 g of LiF was dissolved in 20 mL of 6 m HCl and stirred at room temperature for 10 min. Then, 1 g of Ti_3_AlC_2_ was slowly added into the solution and stirred continuously for 10 min. Subsequently, the mixture was stirred for 24 h at 55 °C to remove the Al layers from Ti_3_AlC_2_. After that, the suspension was centrifuged at 7000 rpm and washed with anhydrous ethanol and deionized water in turn to remove HF until the pH > 5. Finally, the precipitate was dried under vacuum at 60 °C for 10 h and the multi‐layer Ti_3_C_2_T*_x_* was obtained, which was denoted as M‐T.

### Syntheses of Single Layer Ti_3_C_2_T*_x_* Nanosheets

Typically, single‐layer Ti_3_C_2_T*_x_* nanosheets (denoted as S‐T) were synthesized with Li_3_AlF_6_ as an intercalator by means of MAI strategy. First, a certain molar ratio of Li_3_AlF_6_ and 50 mg of M‐T were added into a 50 mL round bottom flask followed by addition of 10 ml EG. The suspension was treated by ultrasound for 10 min to mix evenly. After that, the suspension was put into a modified domestic microwave oven (Galanz, 900 W) and heated for 5 min with 100% output of the power under stirring conditions accompanied with reflux condensation. After cooling to room temperature and stratifying, a black precipitate (the intercalated Ti_3_C_2_T*_x_*, denoted as L‐T) was separated by removing the supernatant. Then, L‐T was dispersed into a certain amount of deionized water and shook well. After ultrasonication for 10 min, a black upper suspension was poured out and collected. Continuously repeated the operation and collected the suspensions until relatively transparent. 1 g of NH_4_HCO_3_ was added into the collected black upper suspensions, which was a colloidal solution of negatively charged single‐layer Ti_3_C_2_T*_x_* nanosheets. After continuously stirring for 10 min, the negatively charged single‐layer Ti_3_C_2_T*_x_* nanosheets were precipitated due to the electrostatically coagulation from NH_4_^+^ ions. The black precipitate was washed with water until the pH ≈ 7 and then a certain amount of dilute sulfuric acid was added to remove the intercalator Li_3_AlF_6_. Subsequently, after washing with water continuously until neutral pH followed by freeze‐drying under vacuum for 48 h, the fluffy black powdery single‐layer Ti_3_C_2_T*_x_* nanosheets with ammonium ions (denoted as NS‐T) adsorbed on surfaces were obtained. Finally, the as‐prepared NS‐T sample was annealed in argon gas at 200 °C for 3 h to remove the adsorbed NH_4_^+^ ions and the final black powdery single layer Ti_3_C_2_T*_x_* nanosheets (donated as S‐T) were obtained.

As contrasts, in addition, LiCl, KCl or KCl+Li_3_AlF_6_ were used for microwave‐assisted intercalation of the above M‐T products under the same other conditions and the intercalation effects were also investigated.

### Preparation of Pd/S‐T Composite Catalyst

Firstly, the surfaces of S‐T were modified by alkalization to increase the number of oxygen‐containing functional groups and improve its hydrophilicity. 0.1 g of KOH was dissolved into 20 mL of water, and then 15 mg of S‐T was added. The mixture was sonicated for 10 min followed by stirring for 1 h. After that, 2.8 mg of PdCl_2_ and 50 mg of CTAB were added in turn and sonicated for 10 min. Then, 5 mL of 0.5 m NaBH_4_ solution was added dropwise. After reaction for 1 h under stirring, the precipitate was separated by centrifugation, washed alternately with deionized water and ethanol for 3 to 4 times, dried by vacuum at 60 °C for 8 h, and finally Pd/S‐T composite catalyst was obtained.

### Hydrogenation of Nitroarenes

5 mg of Pd/S‐T composite catalyst was dispersed into 10 mL of ethanol by sonication and then added into a 25 mL Teflon‐lined autoclave. Next, 1.0 mmol of nitrobenzene was added. After removal of air with H_2_, the autoclave was charged with a H_2_ pressure of 0.5 MPa, and the solution was stirred at 800 rpm for 20 min at 25 °C. After reaction, 1 mmol of ethylbenzene was added as an internal standard. The reaction mixture was analyzed by gas chromatography (GC‐2010 plus, Wonda capillary Column, 30 m × 0.25 mm × 0.25 µm) equipped with a flame ionization detector (FID). The temperature of the gasification chamber was 240 °C, splitting rate was 10:1, the column temperature was 50 °C, the heating rate was 10 °C min^−1^ and the detector temperature was 240 °C. As a contrast, the same experiment was conducted for hydrogenation of nitrobenzene with 3.4 mg of Pd/C (Pd = 10wt%) catalyst under the same conditions.

### Characterization and Calculation

The phase compositions of the samples were determined by a X‐ray diffractometer (XRD, Bruker D8 advance) employing Cu *K*_α_ radiation with 40 kV and 50 mA. The microstructures and elemental distributions of the samples were examined using a scanning transmission electron microscopy (STEM, Talos F200X G2, Thermo Scientific). The morphologies of the samples were observed with a field emission scanning electron microscopy (SEM, SU8010, Hitachi. The thickness of single‐layer Ti_3_C_2_T*_x_* samples was measured using an atomic force microscopy (AFM, SPM‐9700HT). The surface elemental compositions of materials were analyzed by X‐ray photoelectron spectroscopy (XPS, ESCALAB 250Xi). Yield of single layer Ti_3_C_2_T*_x_* nanosheets: *Y* = (*M*_1_/*M*_0_) × 100%, where *M*_1_ is the mass of S‐T obtained by the MAI strategy, *M*_0_ is the mass of M‐T, and *Y* is the yield. The loading amount of Pd was determined by ICP‐OES (iCAP 7400, Thermo Scientific). The surface electronegativity was measured on laser particle size and zeta potential analyzer (Nano ZS90, Malvern).

## Conflict of Interest

The authors declare no conflict of interest.

## Supporting information

Supporting Information

## Data Availability

The data that support the findings of this study are available in the Supporting Information of this article.
