# Supplementary material for: Microwave‐Assisted Efficient Intercalation for Fast Fabrication of High‐Quality and Large‐Size Single‐Layer Ti3C2T x Nanosheets
Source: Adv Sci (Weinh). 2024 Jul 2;11(33):2405686. doi: 10.1002/advs.202405686 (PMC11434240; doi:10.1002/advs.202405686)
Supplement: Supplementary file 1 — Supporting Information [file ADVS-11-2405686-s001.docx]

Supporting Information

**Microwave-assisted efficient intercalation for fast fabrication of high-quality and large-size single layer Ti_3_C_2_T_x_ nanosheets**

Yitian Zhong, Qixi Zhang, Shuling Lan, Haosheng Feng, Yanxi Zhao, Qin Li, Xianghong Li, Tao Huang*

Key Laboratory of Catalysis and Energy Materials Chemistry of Ministry of Education, College of Chemistry and Materials Science, South-central Minzu University, 430074, China

*Corresponding author: huangt208@163.com

Figures


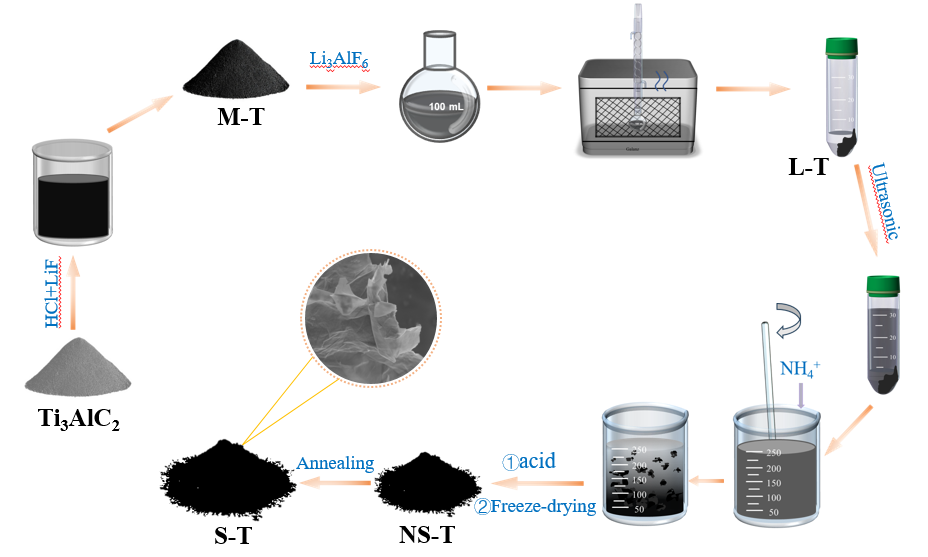


**Figure S1**. The experimental procedure of the S-T preparation.


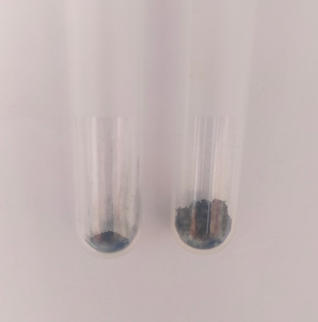

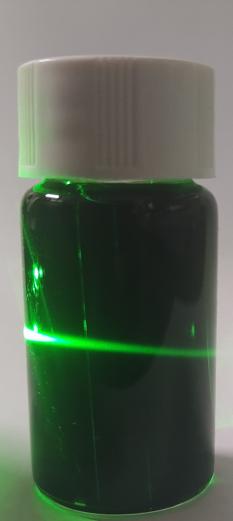

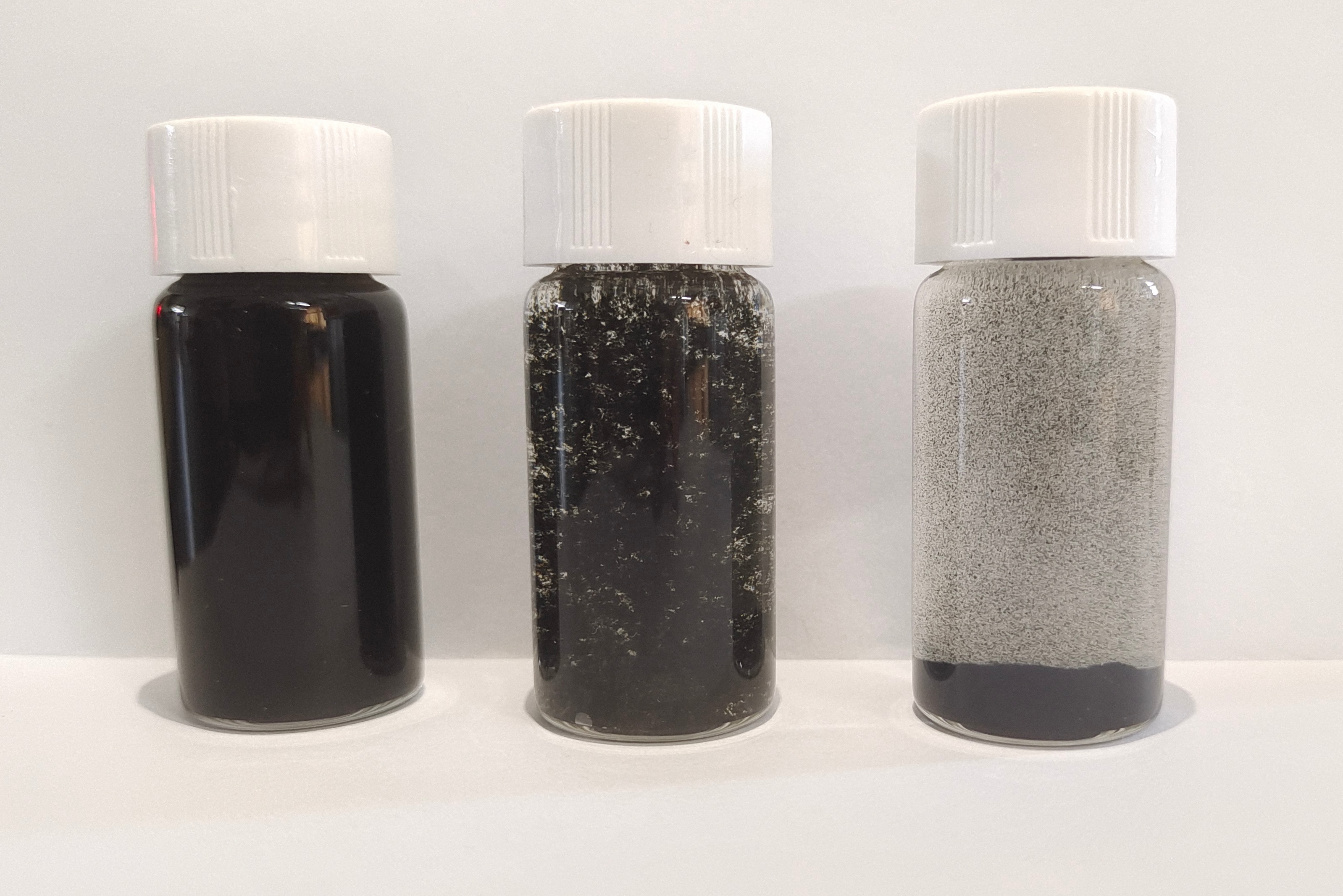

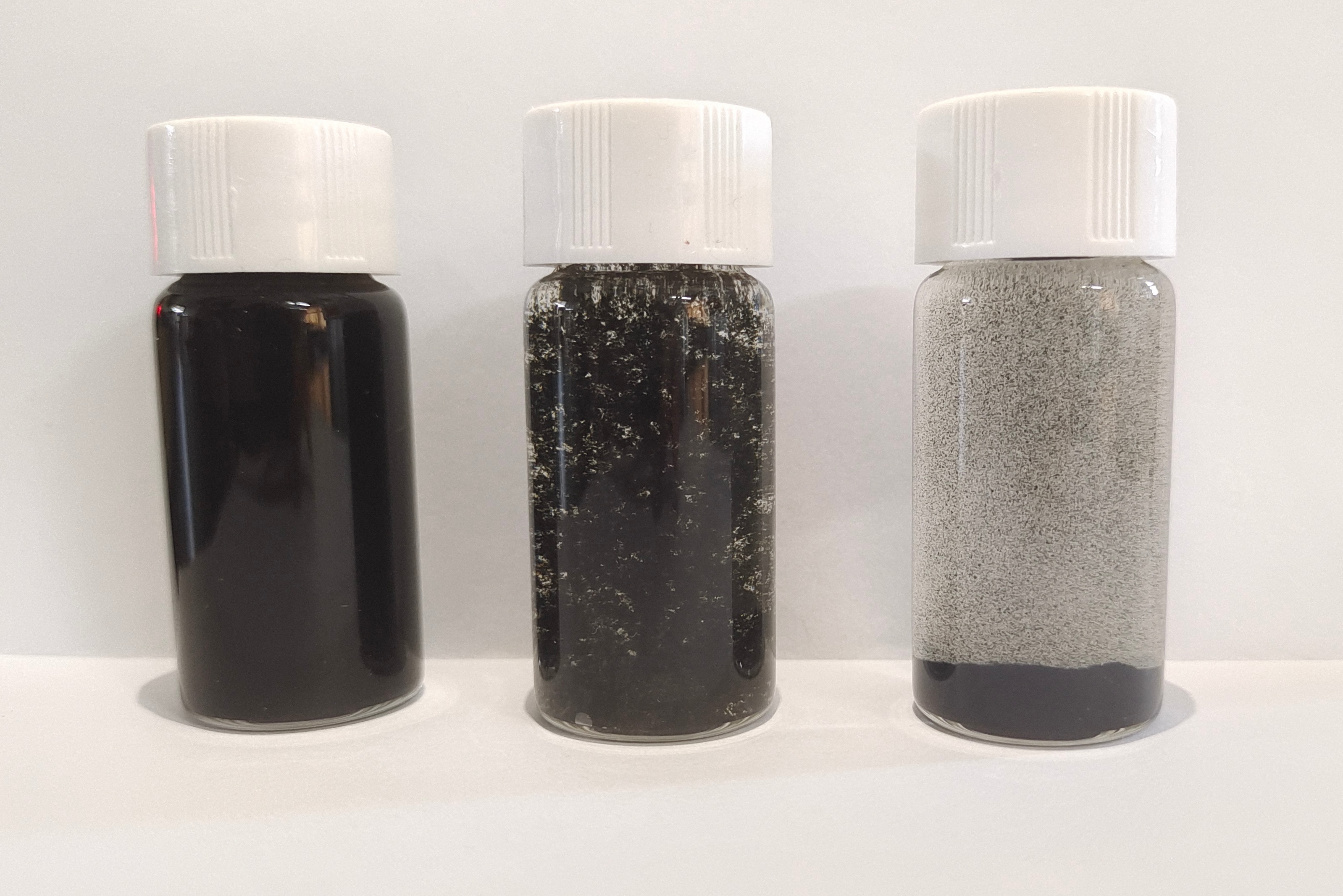

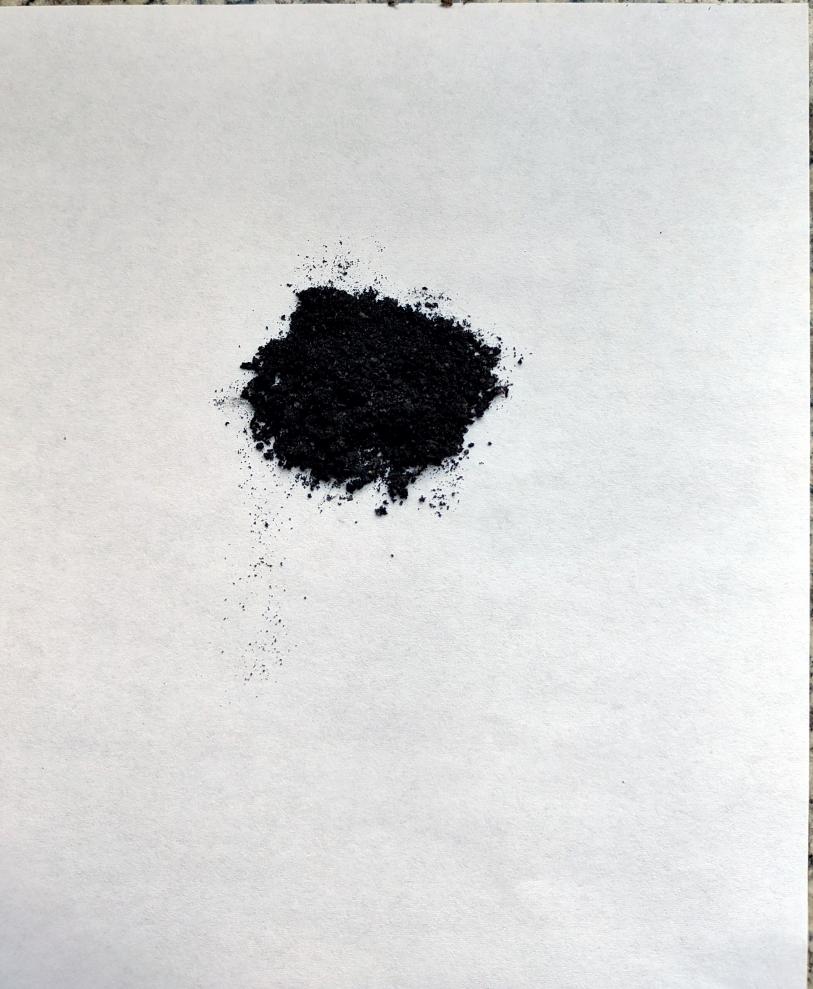


**d**

**S-T**

50 mg M-T 25 mg S-T

**(d)**

**(a)**

**(c)**

**(b)**

**d**

**Figure S2**. (a)Tyndall effect of the suspension with single-layer Ti_3_C_2_T_x_ nanosheets (L-T); (b) The black floccules of L-T after adding NH_4_^+^; (c) The precipitate after 24 h of electrostatically deposition with NH_4_^+^; (d) S-T powder. The inset of (d) shows a volume comparison for 25 mg S-T and 50 mg M-T.

**Figure S3.** The effect of the amount of intercalating agent on the yield of S-T.





**Figure S4**. The as-prepared multi-layer Ti_3_C_2_T_x_ after etching of Ti_3_AlC_2_.

**Figure S5**. XRD pattern of multi-layer Ti_3_C_2_T_x_ after etching of Ti_3_AlC_2_.





(a)





(b)

**Figure S6**. SEM images of uncleaned accordion shaped multi-layer Ti_3_C_2_T_x_ (a) and M-T sample (b) without adding any other intercalator after microwave irradiation for 5 min.


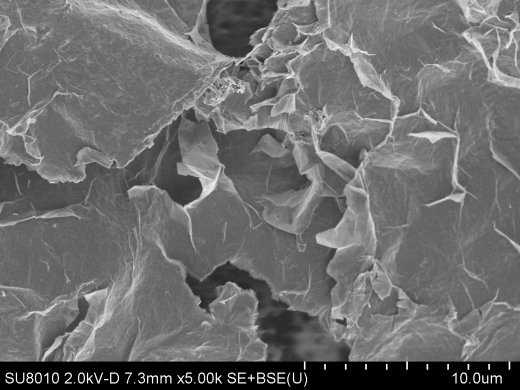

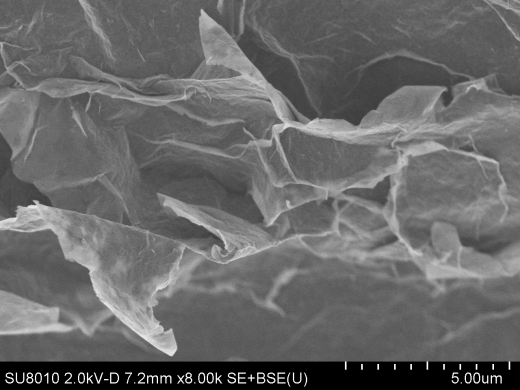


(b)

(a)


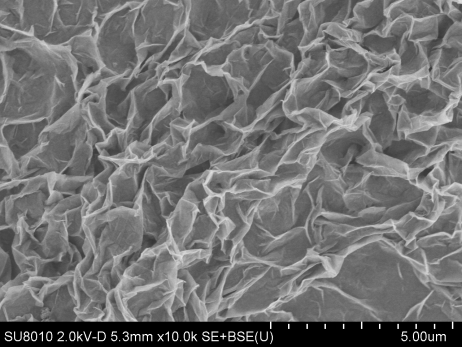

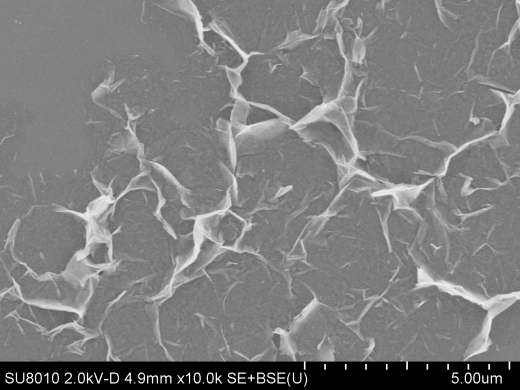


(d)

(c)

**Figure S7**. SEM images of S-T samples with a large size over 20µm within planar extension. (a) and (b) solid powder; (c) and (d) dispersed sample in water.


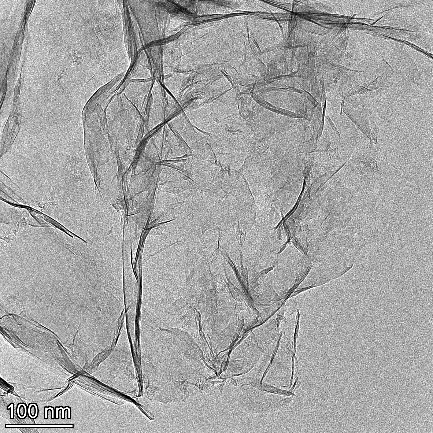

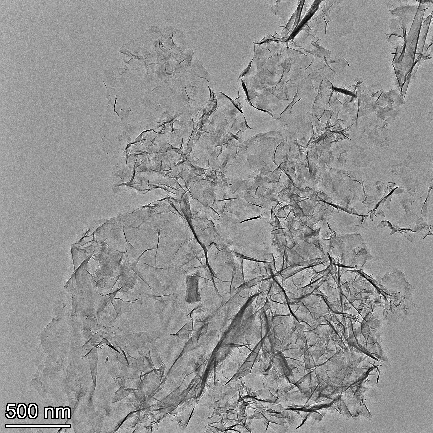


**Figure S8**. TEM images of S-T samples.

**Figure S9**. The XRD patterns of the products obtained using different intercalators under microwave irradiation for 5 min followed by ultrasonic exfoliation, in which no intercalator, LiCl or KCl, KCl+Li_3_AlF_6_ and Li_3_AlF_6_ was used, respectively.






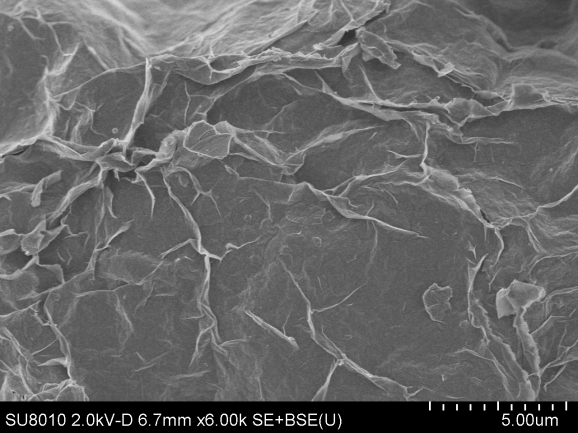

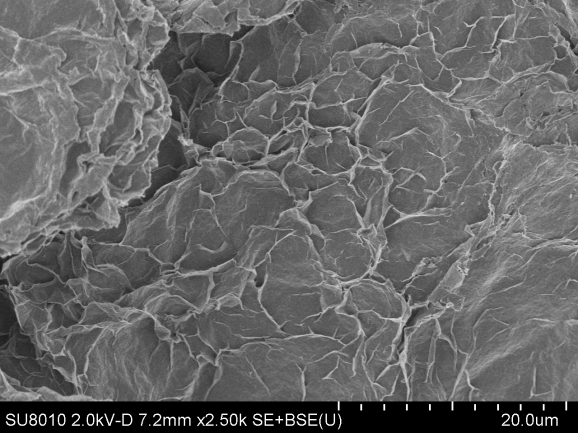


(a)

(b)

(d)

(c)

**Figure S10**. SEM images of the samples obtained with different intercalators. (a) LiCl; (b) KCl; (c) KCl+Li_3_AlF_6_; (d) Li_3_AlF_6_.


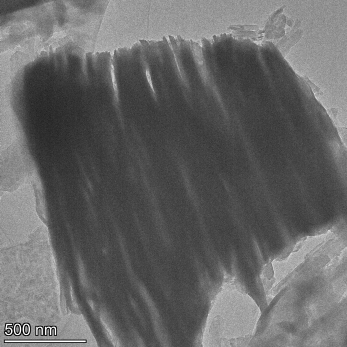

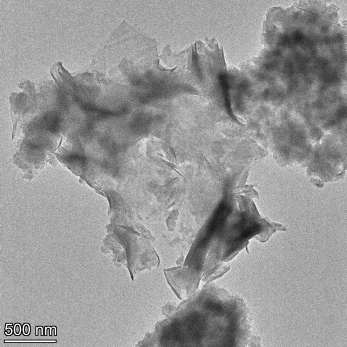


(a)

(b)

(c_3_)

(c_2_)

(c_1_)


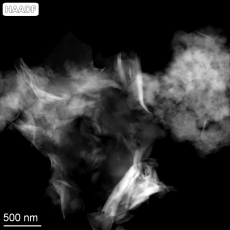

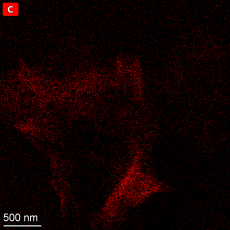

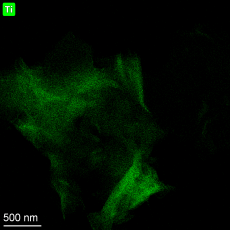


**Ti**

**C**

(c_6_)

(c_4_)

(c_5_)


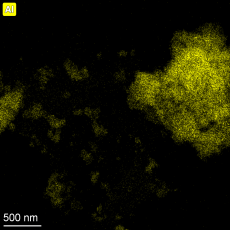

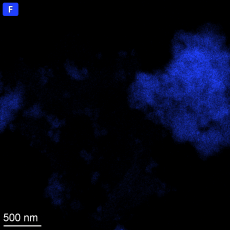

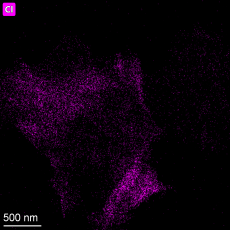


**Al**

**Cl**

**F**


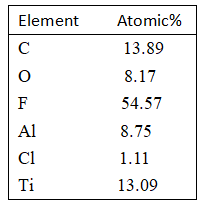


(d)


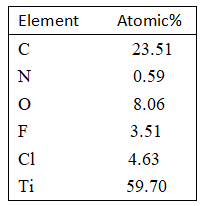


(e)

**Figure S11**. (a) and (b) TEM images of M-T and L-T, respectively; (c_1_) HAADF-STEM image of L-T; (c_2_-c_6_) EDS mappings of L-T, in which C, Ti, Al, F and Cl were presented; (d) EDS spectrum of L-T sample; (e) EDS spectrum of NS-T sample.

(b)

(a)


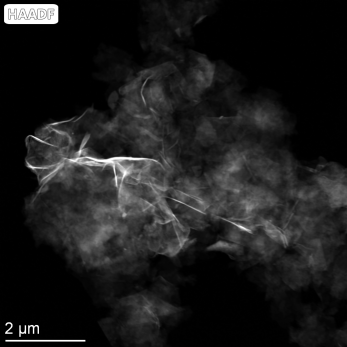

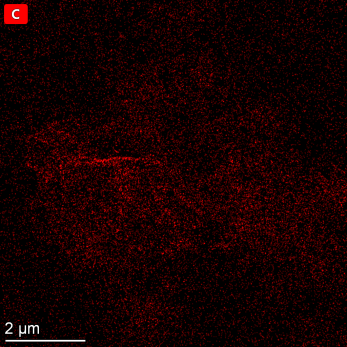

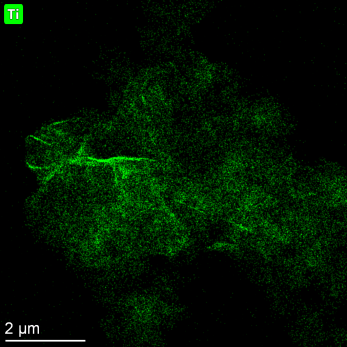


**C**

**Ti**

(d)

(e)

(f)

(c)


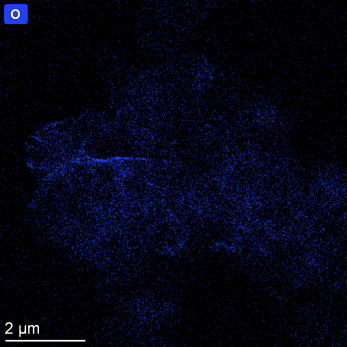

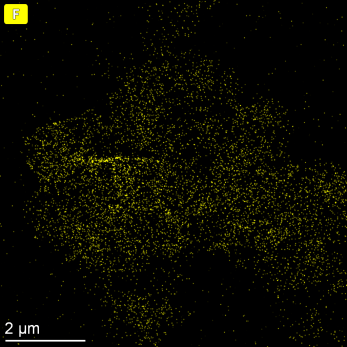

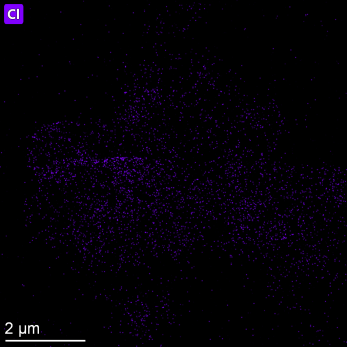


**O**

**Cl**

**F**

(g)


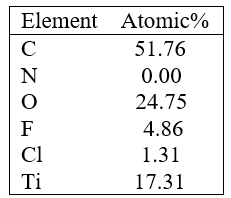


**Figure S12**. EDS mappings of S-T sample. (a) HAADF-STEM image; (b)-(f) C, Ti, O, F and Cl elements were presented, respectively; (g) EDS spectrum of S-T sample.

**Figure S13**. Zeta potential measurements for Li_3_AlF_6_, M-T and L-T.


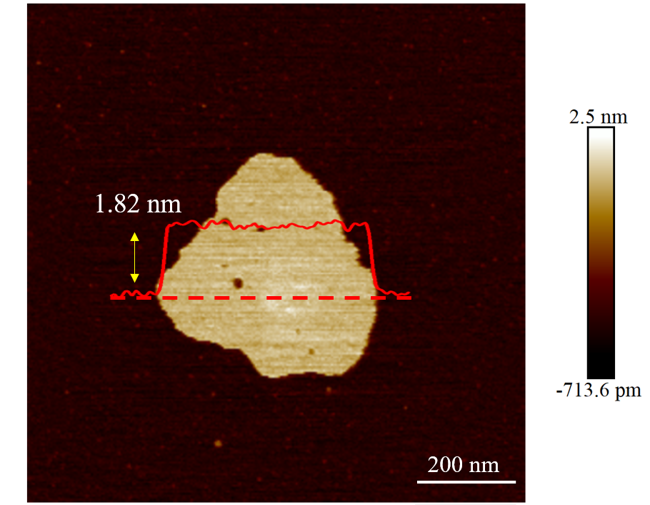


(a)


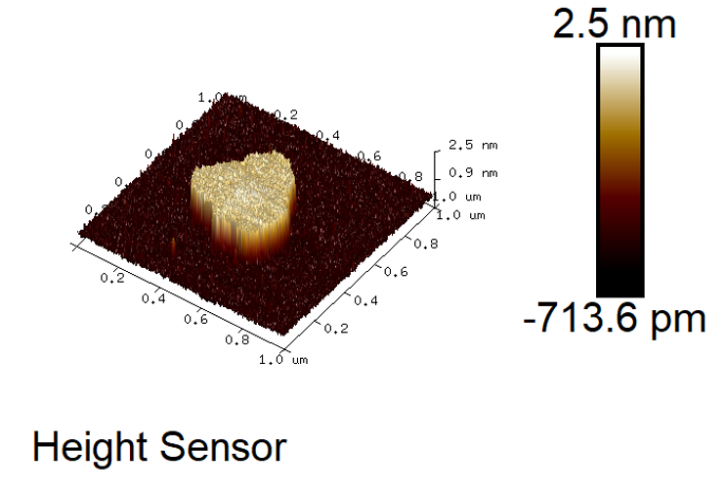


(b)

**Figure S14**. AFM images of a monolayer Ti_3_C_2_T_x_ nanosheet. (a) 2D AFM image ; (b) 3D AFM image.


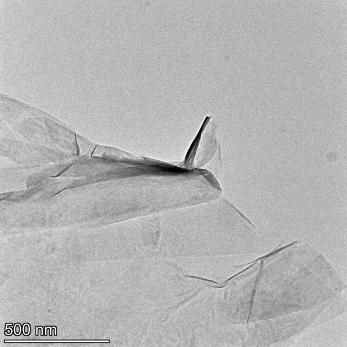

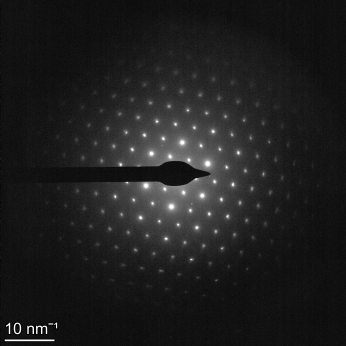


(b)

(a)

**Figure S15.** STEM (a) and the corresponding SAED pattern (b) of a monolayer Ti_3_C_2_T_x_ nanosheet.

**Figure S16.** The full-scale XPS spectra of S-T sample.

**(b)**

**(a)**

**(d)**

**(c)**

**Figure S17.** XPS analyses of S-T samples. (a) Ti 2p region; (b) C 1s region; (c) O 1s region; (d) F 1s region.


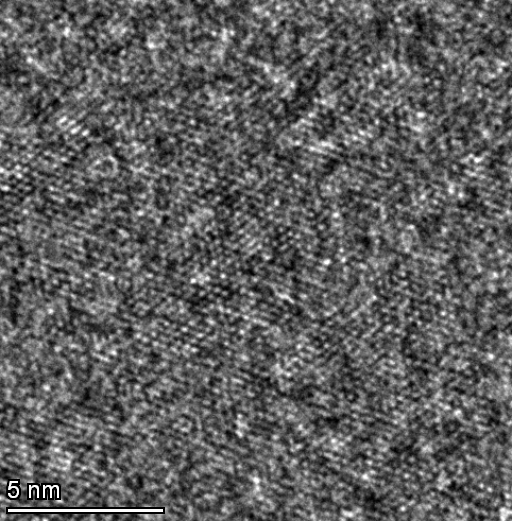


**V_Ti_**

**Figure S18**. HRTEM image of S-T with Ti vacancy defects.


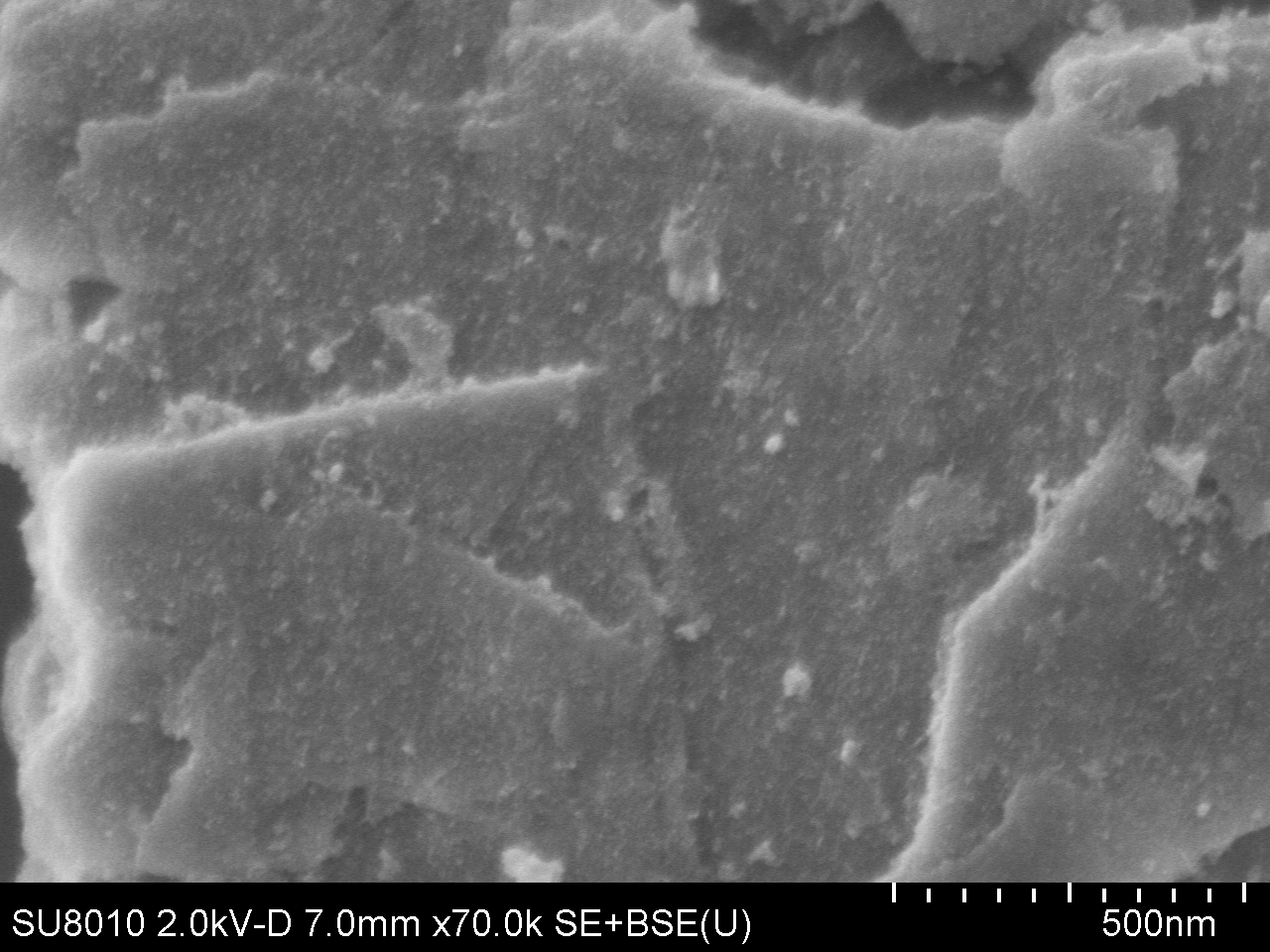


**Figure S19**. SEM image of Pd/S-T composite.


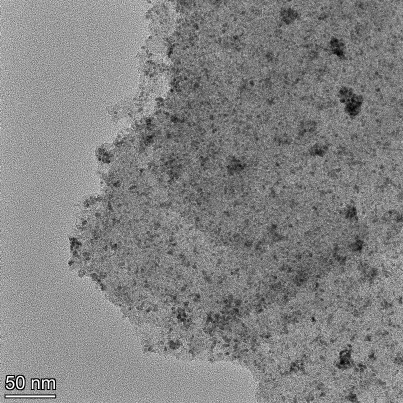

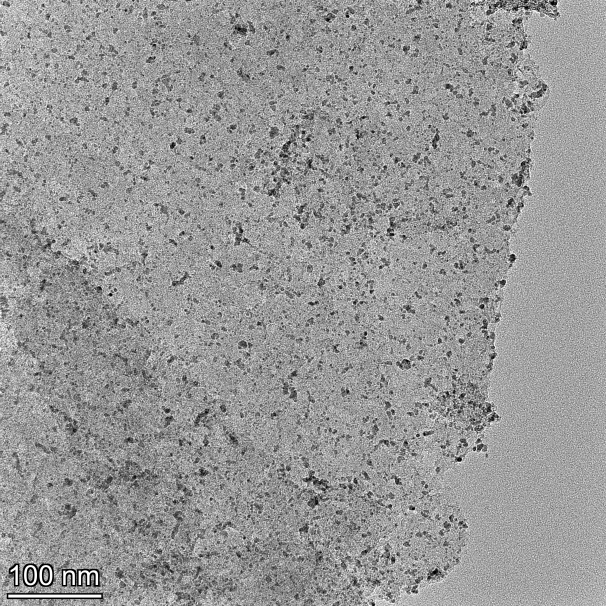


**(b)**

**(a)**

**(h)**

**C**

**Pd**

**Ti**

**Figure S20**. (a) and (b) TEM images of Pd/S-T with different scales. The inset of (b) shows the size distribution of Pd nanoparticles.

**(c)**

**(d)**

**(f)**

**(e)**

**(b)**

**(a)**

**Figure S21.** XRD and XPS analyses of Pd/S-T samples. (a) XRD pattern; (b) full scale XPS; (c) Pd 3d region; (d) Ti 2p region; (e) C 1s region; (f) O 1s region.

**Figure S22.** Effect of reaction time on hydrogenation performance of nitrobenzene over Pd/S-T catalyst. Reaction conditions: Nitrobenzene, 1 mmol; Pd/S-T catalyst, 5 mg; Ethanol, 10 mL; 25 °C; 20 min.

**Figure S23.** GC analysis of nitrobenzene hydrogenation with Pd/S-T and Pd/C catalysts. Reaction conditions: Nitrobenzene, 1 mmol; 5 mg of Pd/S-T or 3.4 mg of Pd/C (Pd=10 wt %) catalyst; Ethanol, 10 mL; 25 °C; 20 min.

**Table S1.** Chemoselective hydrogenation of several nitroarenes.

| Entry | Substrate | Product | Pd/S-T  (mg) | Time (min) | Conversion  (%) | Selectivity(%) |
| --- | --- | --- | --- | --- | --- | --- |
| 1 |  |  | 5 | 20 | 100 | 100 |
| 2 |  |  | 5 | 30 | 100 | >99 |
| 3 |  |  | 5 | 30 | 100 | >99 |
| 4 |  |  | 10 | 90 | 100 | >99 |
| 5 |  |  | 10 | 120 | 97.9 | 95.7 |

The reaction conditions were as follows: nitroarenes, 1 mmol; H_2_ pressure, 0.5 MPa; ethanol, 10 ml; 25 ℃

**Figure S24.** GC analyses of hydrogenation products of various nitroarenes over Pd/S-T catalyst. The reaction conditions were as follows:Nitrobenzene, 1 mmol; Pd/S-T catalyst, 5 mg; H_2_ pressure, 0.5 MPa; Ethanol, 10 ml; 25 °C; 20 min.

**Figure S25.** GC analyses of nitrobenzene hydrogenation with H_2_ for recycling test over Pd/S-T catalyst. The reaction conditions: Nitrobenzene, 1 mmol; Pd/S-T catalyst, 5 mg; H_2_ pressure, 0.5 MPa; Ethanol, 10 ml; 25 °C; 20 min. Internal standard: ethylbenzene.


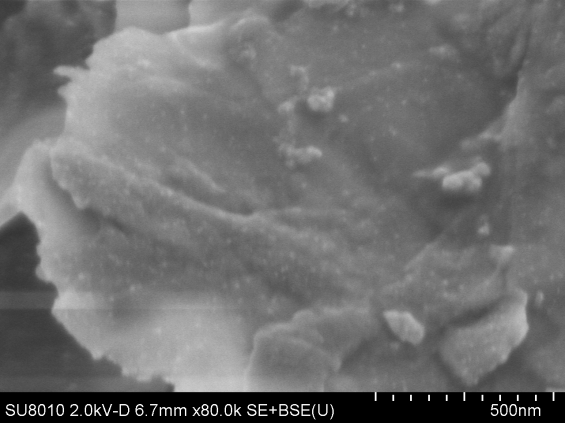


**(a)**


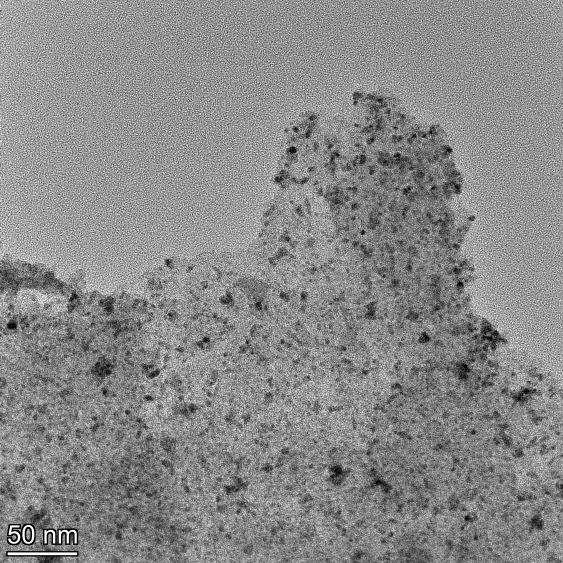


**(b)**

**(c)**

**Figure S26.** SEM (a) and TEM (b) images, and particle size distribution (c) of Pd/S-T catalyst after 5 cycles. The reaction conditions: Nitrobenzene, 1 mmol; Pd/S-T catalyst, 5 mg; H_2_ pressure, 0.5 MPa; Ethanol, 10 ml; 25 °C; 20 min.


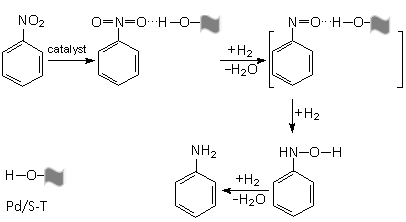


**Figure S27.** Mechanism of nitrobenzene hydrogenation over Pd/S-T catalyst.
